# Addressing the Challenge of Assessing Physician-Level Screening Performance: Mammography as an Example

**DOI:** 10.1371/journal.pone.0089418

**Published:** 2014-02-21

**Authors:** Elizabeth S. Burnside, Yunzhi Lin, Alejandro Munoz del Rio, Perry J. Pickhardt, Yirong Wu, Roberta M. Strigel, Mai A. Elezaby, Eve A. Kerr, Diana L. Miglioretti

**Affiliations:** 1 Department of Radiology, University of Wisconsin School of Medicine and Public Health, E3/311 Clinical Science Center, Madison, Wisconsin, United States of America; 2 Department of Biostatistics and Medical Informatics, University of Wisconsin, Madison Wisconsin, United States of America; 3 Department of Medical Physics, University of Wisconsin, Madison, Wisconsin, United States of America; 4 Veterans Affairs Center for Clinical Management Research, VA Ann Arbor Healthcare System, Ann Arbor, Michigan, United States of America; 5 Department of Internal Medicine, University of Michigan Medical School, Ann Arbor, Michigan, United States of America; 6 Group Health Research Institute, Group Health Cooperative, Seattle, Washington, United States of America; Stanford University School of Medicine, United States of America

## Abstract

**Background:**

Motivated by the challenges in assessing physician-level cancer screening performance and the negative impact of misclassification, we propose a method (using mammography as an example) that enables confident assertion of adequate or inadequate performance or alternatively recognizes when more data is required.

**Methods:**

Using established metrics for mammography screening performance–cancer detection rate (CDR) and recall rate (RR)–and observed benchmarks from the Breast Cancer Surveillance Consortium (BCSC), we calculate the minimum volume required to be 95% confident that a physician is performing at or above benchmark thresholds. We graphically display the minimum observed CDR and RR values required to confidently assert adequate performance over a range of interpretive volumes. We use a prospectively collected database of consecutive mammograms from a clinical screening program outside the BCSC to illustrate how this method classifies individual physician performance as volume accrues.

**Results:**

Our analysis reveals that an annual interpretive volume of 2770 screening mammograms, above the United States’ (US) mandatory (480) and average (1777) annual volumes but below England’s mandatory (5000) annual volume is necessary to confidently assert that a physician performed adequately. In our analyzed US practice, a single year of data uniformly allowed confident assertion of adequate performance in terms of RR but not CDR, which required aggregation of data across more than one year.

**Conclusion:**

For individual physician quality assessment in cancer screening programs that target low incidence populations, considering imprecision in observed performance metrics due to small numbers of patients with cancer is important.

## Introduction

Metrics used to evaluate the quality of a cancer screening program often parallel performance characteristics of randomized controlled trials (RCTs) that have demonstrated a mortality benefit and have thereby established the efficacy of the test–typically detection rates and false positives [1]. For screening tests that require physician expertise, like mammography [2], [3] and colonoscopy [4], [5], variability of practice has been observed and undoubtedly compromises the quality and efficacy of the overall program. There is a large corpus of literature demonstrating the difficulties in accurately identifying outliers, particularly on the individual physician level [6]–[12]. However, policy-makers and health systems are increasingly requiring reporting of screening performance on the physician level, for example in United States (US), via the Physician Quality Reporting System [13]. Accurate performance assessment in cancer screening is particularly challenging because disease incidence is low. An attractive method of identifying outlier physicians, if available, is comparison to an absolute cut-off level generated from national benchmarks or guidelines [6], [14]. However, one caveat to this method of performance evaluation is that observed performance values may be imprecise if generated from small (and therefore highly variable) populations inherent in low volume practice [6], [15].

Mammography screening may be the best studied screening test, perhaps due to rigorous performance of RCTs, development of large, high-quality, population-based data sets and subsequent quality legislation. For these reasons, we use mammography as our example. The Mammography Quality Standards Act (MQSA), established in 1992 in the US, requires each mammography facility to have a medical audit system for follow-up and outcome analysis but stops short of requiring that physicians meet specific performance criteria [15]. Other nations with breast cancer screening programs have a spectrum of systems of quality assurance. However most systems use at least two metrics, cancer detection rate (CDR) and recall rate (RR), to compare and classify individual physician performance for mammography screening (individual physician CDR and RR measurements will be henceforth called “observed performance values”). Recommended screening mammography performance ranges (henceforth called “benchmarks”) have also been established, refined, and documented in the literature [15]–[19] by using population-based reference distributions [19] or consensus methods [15]. For example, Carney et al. published consensus levels of minimally acceptable performance for CDR (above 2.5/1000) and RR (between 5 and 12%) and found that 28.4% of a community-based sample of US interpreting physicians the National Cancer Institute Breast Cancer Surveillance Consortium (BCSC) were below this benchmark for CDR and 49.1% were outside the range for RR [15]. CDR and RR are closely related and should be considered together because higher true positive rates (estimated by CDR) are generally correlated with higher false positive rates (estimated by RR) [20]–[22].

Observed performance values for many physicians in the BCSC were based on a small number of mammograms, especially those performed on women with cancer, possibly leading to misclassification of some physicians based on imprecise estimates. The volume of interpreted mammograms directly influences the size of the confidence interval around observed performance values and these confidence intervals should be considered in the evaluation of individual physicians. Although interpretative volume has been recognized as a source of inaccuracy when assessing performance benchmarks historically [15], judgments based on observed performance values have not considered volume [19]. We develop a method for asserting adequate or inadequate screening performance or identifying when more data (higher volume) is required for individual physician-level performance evaluation, and demonstrate this method for screening mammography.

## Materials and Methods

Our Institutional Review Board did not require that this HIPAA-compliant, retrospective quality-assurance project involve informed consent. We define CDR and RR benchmarks based on the BCSC reference distribution derived from seven mammography registries in the US [15], [19].

Cancer detection rate (CDR) is the number of true positive screening mammograms divided by the total number of screening mammograms performed [23]. In the BCSC, CDR for the middle 80% of physicians ranges from 2.4/1000 to 7.0/1000 with a median of 4.4/1000 [19]. Higher CDR is always desirable with an upper limit constrained by the incidence of disease. Low CDR values typically reveal suboptimal performance. For clarity, we define the **benchmark threshold** as a limit (selected based on a reference distribution–the10^th^ or 90^th^ percentile of the BCSC, in our case) that the confidence interval (selected based on the desired level of confidence–95%, in our case) of an individual physician’s performance value must not overlap in order to be deemed adequate. For CDR, we define the benchmark threshold as the 10^th^ percentile of the BCSC reference distribution, which is 2.4/1000.

Recall rate is the number of positive screening mammograms (true positive+false positive) divided by the total number of screening examinations interpreted [23]. Of note, the lower limit of total positives should ideally be characterized by the trade-off between true positives and total positives (CDR vs. RR). In other words, a low RR is only “bad” if it results in low CDR. We therefore focus on detecting RRs that are too high, assuming a low RR that is “bad” will be identified by a low CDR. The middle 80% of BCSC physicians had recall rates between 4.4% and 16.8% with a median of 9.7% [19]. For RR, we define the benchmark threshold as the 90^th^ percentile of the BCSC reference distribution, which is 16.8%.

We divide screening interpretive performance into 3 categories: 1) met benchmark “with confidence” (**adequate performance**) meaning all the values in the confidence interval for the individual performance value meet or exceed the benchmark threshold, 2) **uncertain performance** meaning the 95% confidence interval overlaps the benchmark threshold, and 3) did not meet benchmark “with confidence” (**inadequate performance**) meaning that all the values in the confidence interval for the individual performance value fall short of the benchmark threshold.

### Clinical Data

In order to validate our approach, we felt it important to analyze our framework on a practice not included in the BCSC population. Therefore, we analyzed consecutive screening mammograms performed at our institution (also in the US) from 1/1/2006 to 12/31/2008. All mammographic findings were prospectively described and recorded (at the time of mammography interpretation) by the interpreting physician using the Breast Imaging Reporting and Data System (BI-RADS) assessment categories–Table 1. We included physicians who read more than 480 mammograms per year (corresponding to the volume mandated by MQSA [24]) in the 3 years that we analyzed. Four physicians met our inclusion criteria; all were MQSA certified with 5–15 years of experience and 3 were fellowship trained.

**Table 1:** BI-RADS* final assessment categories with associated recommendation.

Category	Definition	Recommendation	
0	Needs additional imaging evaluation	Additional imaging	
1	Negative	None (routine mammography)	
2	Benign finding	None (routine mammography)	
3	Probably benign finding	Short-interval follow-up (6 months)	
4	Suspicious abnormality	Biopsy	
5	Highly suggestive of malignancy	Biopsy	

*BI-RADS Version 4 [22].

Since demographic factors like age, family history of breast cancer, personal history of breast cancer, breast density, and comparison with prior mammography [25]–[27] have repeatedly been shown to influence clinical outcomes for screening mammography, we measured these parameters to understand the underlying demographics of our population and to compare to the BCSC reference population [19].

### Outcomes

We calculated cancer detection rate and recall rate as per BI-RADS methodology (also used in the BCSC data) on an individual physician level [19], [23]. Our institutional Cancer Center Registry serves as the reference standard for each mammography examination [28]. A positive mammogram (recall) is a mammogram with an initial BI-RADS assessment of 0, 4, or 5 based on routine screening views. A detected cancer is a diagnosis of invasive breast cancer or ductal carcinoma in situ (DCIS) within 12 months following a positively interpreted screening mammogram examination.

### Statistical Analysis

We propose a graphical method to illustrate the classification of performance into three categories (adequate, uncertain, and inadequate) based observed performance values and interpretive volume, for a given benchmark threshold. Performance categories are defined by first calculating a 95% confidence interval (CI) for the observed performance and then assessing whether the benchmark threshold lies above, within, or under the 95% CI. We used the Wilson score confidence interval method with continuity correction [29] to compute two-sided confidence intervals for the binomial proportions CDR and RR. We derived equations (Appendix) for the minimum (or maximum) performance value that provides 95% confidence that a physician is performing adequately for any specified volume. From these graphs, we obtained the screening mammography volume required to assert with confidence that achievement of the benchmark median equates to adequate performance [19]. Since the CDR is a small proportion and may thus be imprecisely estimated, we obtained coverage probabilities small proportions and may thus be imprecisely estimated, we obtained coverage probabilities [30] to assess any possible discrepancy between the nominal confidence interval and the actual coverage probability–details are covered in the Appendix and illustrated graphically in the Appendix figures (4a and 4b). Statistical computations were done in R 2.15.2 [31] with the binom.coverage() function with the binom package [32].

## Results

**Figure 1:**
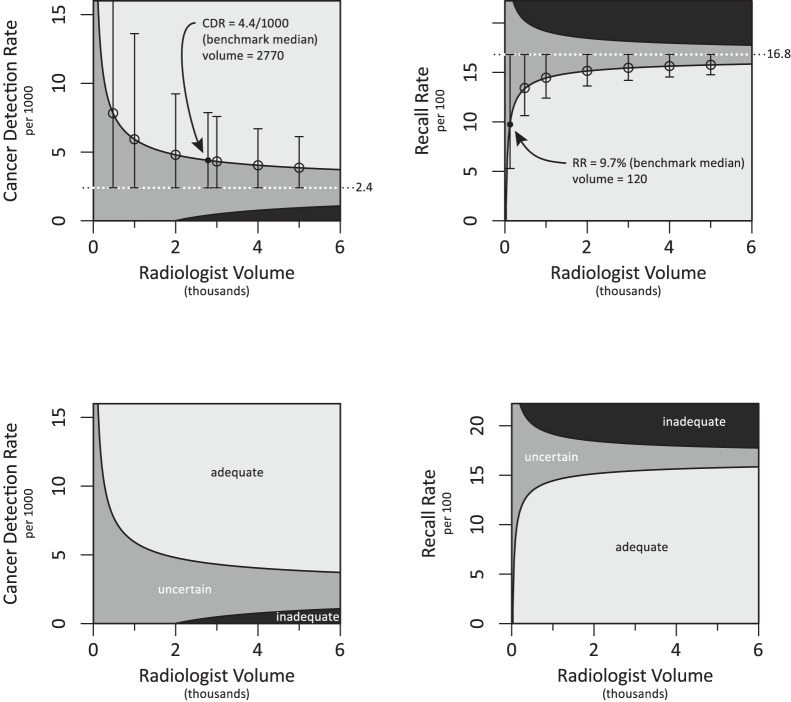
Defining adequate performance based on volume. Plots demonstrate our method for constructing curves by using the benchmark threshold as the limit of 95% confidence based on volume: (A) CDR performance levels are established using 2.4 as the lower boundary for 95% CI of adequate performance (CIs shown) and the upper boundary for inadequate performance (CIs not shown). This methodology shows (indicated with a black dot) that a volume of 2770 is required to confidently assert the CDR benchmark median of 4.4/1000 is adequate; (B) RR performance levels are established using 16.8 as the upper boundary for 95% CI of adequate (CI shown) and inadequate (CI not shown) performance. A volume of 120 (indicated with a black dot) is required to confidently assert the RR benchmark median of 9.7% is adequate. Plots define regions of adequate, uncertain, and inadequate performance for (B) CDR and (D) RR.

Graphical representations of the observed performance values required to provide 95% confidence of adequate or inadequate performance given our selected benchmark threshold and a range of volumes are shown in Figures 1a for CDR and 1b for recall rate. A volume of 2770 screening mammograms is required to confidently assert that a CDR of 4.4/1000 (the benchmark median) equates to adequate performance (Figure 1a–value shown as black circle denoted by an arrow). At this level of performance and volume, the lower bound of the 95% confidence interval meets the benchmark threshold of 2.4/1000, as defined in the methods. The volume required to confidently assert that the benchmark median for RR (9.7%) is much lower at 120 screening mammograms (Figure 1b– value shown as black circle denoted by an arrow).

During the 3 year time period we analyzed clinical data (from outside the BCSC), 30,363 screening mammograms were performed for 18,069 women. We compare our study population to the BCSC population in Table 2. The mean age of our population was 56.5 (range = 22–96; standard deviation = 11.12) years. Similar to the BCSC population, the majority of screening examinations, 83.5% (27,389 of 32,793) were performed in women within the typical screening age range of 40–69 years with the minority of women outside this range: 2.4% (795 of 32,793) younger than 40 years and 13.5% (795 of 32,793) older than 70 years.

**Table 2:** Distribution of study population.

		*No Cancer*	*(%)*	*Cancer*	*(%)*	*Total*	*(%)*	*Compare (%)* *
***Number of Mammograms***								
***Age Groups***								
	<30	20	0.1	0	0.0	20	0.1	0.1
	30–39	727	2.4	2	1.2	729	2.4	4.7
	40–49	8205	27.2	24	14.8	8229	27.1	29.3
	50–59	10,339	34.2	45	27.8	10,384	34.2	28.9
	60–69	6796	22.5	52	32.1	6848	22.6	19.1
	70–79	3132	10.4	26	16.0	3158	10.4	13.6
	>80	982	3.3	13	8.0	995	3.3	4.2
***Family History of Breast Cancer***								
	Yes	5818	19.3	46	28.4	5864	19.3	15.2
	No	23,775	78.7	114	70.4	23,889	78.7	84.8
	Unknown	608	2.0	2	1.2	610	2.0	17.4
***Personal History of Breast Cancer***								
	Yes	3071	10.2	74	45.7	3145	10.4	6.3
	No	27,130	89.8	88	54.3	27,218	89.6	93.7
***Comparison films available***								
	Yes	24,484	81.1	143	88.3	24,627	81.1	89.2
	No	5717	18.9	19	11.7	5736	18.9	10.8
***Self-reported symptoms***								
	Yes	1132	3.7	21	13.0	1153	3.8	3.6
	No	29,069	96.3	141	87.0	29,210	96.2	96.4

*According to Rosenberg, et al. [19].

**Figure 2:**
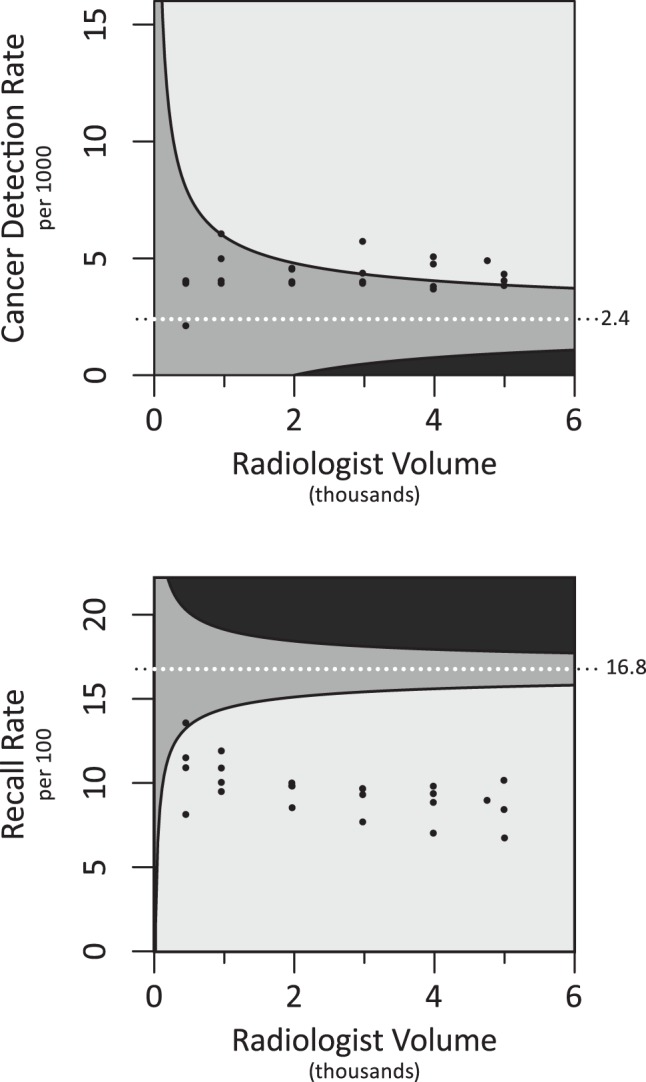
Individual physician performance assessment based on volume. Plots of (A) CDR and (B) RR for the 4 included radiologists at 6 volumes from 500 examinations (then at 1000 and subsequently 1000 exam increments) to the maximum volume read over the 3 years or 5000 total (whichever was least).

Based on this clinical data we analyzed both CDR and RR over three consecutive years. The average yearly volume for the four included physicians was 1918 screening mammograms per year per physician. Plotting observed performance values as volume increases (Figure 2a) demonstrates that below approximately 3000 mammograms, observed CDR performance values resided in the uncertain region because confidence intervals consistently overlap the benchmark threshold. However, as volume increased, all physicians succeeded in achieving a performance value in the adequate range. On the other hand, observed RR performance values quickly settled in the adequate range (Figure 2b).

**Figure 3:**
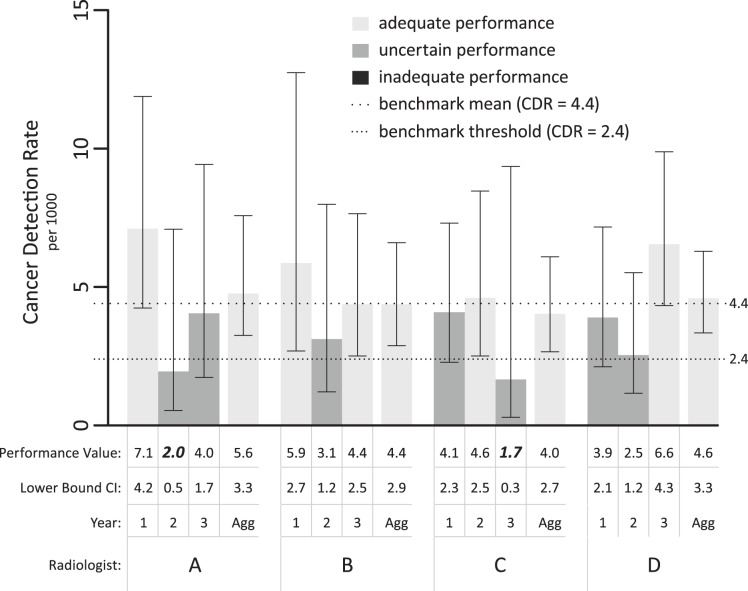
Annual observed performance values as compared to aggregated data. Annual CDR for each individual radiologist are shown on this bar graph with performance values and lower bound 95% CI summarized below the bar graph. The fourth bar for each physician represents performance over the 3 years of the study period aggregated (“Agg”) into a consolidated performance metric. Performance values in th first row in italics and bold represent performance values that would be characterized as inadequate using previously published benchmark thresholds.

Analysis of clinical data from one non-BCSC practice demonstrates that physicians often appear to be underperforming if a single year is viewed in isolation. Out of 12 annual measures of CDR (three for each physician), only 5 demonstrated adequate performance and 7 were in the uncertain range (Figure 3). Furthermore, each physician had at least one annual observed performance value below the benchmark median of 4.4/1000 (n = 9) and half (2 of 4) of the physicians had an annual observed performance value below the level defined for adequate CDR performance in the literature, 2.5/1000 [15] and the benchmark threshold 2.4/1000 [19] (Figure 3–physician A, year 2 and physician C, year 3). However, all 4 physicians showed adequate performance in at least one year and, most importantly, showed adequate performance when all three years were aggregated.

## Discussion

A physician performing a cancer screening test is expected to have a high detection rate while simultaneously maintaining a low false positive rate in order to reap the mortality benefits of early detection while simultaneously minimizing harms. Variability of practice diminishes screening program efficacy [2]–[5], [33] and powerfully motivates physician-level performance evaluation and quality improvement initiatives. However, due largely to low disease incidence, performance diverging from benchmarks may reflect either poor performance or stochastic variation; therefore, without considering volume and variability, substantive rates of physician misclassification is a real risk.

We use mammography with associated national benchmark data (the BCSC reference distribution) as an example to establish combinations of volume and performance that are adequate with certainty, inadequate with certainty, or uncertain and thus require more data. We found that much larger volumes are required to confidently classify physicians based on CDR vs. RR; given cancer detection is a much rarer event than recall. For physicians with observed performance values at the benchmark median, volumes of 2770 screening mammograms for CDR compared to only 120 screening for RR are required to confidently assert their performance is adequate. Importantly, below this volume, physicians must have observed performance values above the benchmark median to confidently assert adequate performance. The average annual screening interpretive volume for a large sample of physicians in the US was 1777 mammograms [34] in agreement with the average of 1918 screening mammograms per year, per physicians in our practice; both substantially less than the 2770 required for robust CDR estimates. However, recommended volumes in other programs like the National Health Service Breast Cancer Screening Program (with a threshold annual volume of 5000 cases) surpasses this level. [35].

By applying our method to physicians outside the BCSC, we find that assessing annual observed performance values to judge CDR for screening mammography without considering volume (i.e. variability) is perilous, because observed measures for individual physicians may fall below the benchmark threshold by chance in a given year. In fact, this occurred for two out of four physicians (half of our non-BCSC sample) when annual performance values were viewed without considering their confidence intervals, despite adequate performance when larger volumes for the same physicians were aggregated (thereby decreasing the variability of observed performance values). Based on established benchmark thresholds in the literature, (e.g. 2.5/1000 [15]) applied to the observed performance measures without considering variability in these measures (i.e., the confidence intervals), these annual observed performance measures might have triggered quality improvement initiatives, possibly unnecessarily. On the other hand, pooling data over time for more precise estimates may generate observed performance values that are less reflective of current skills. Future investigation on this topic will hopefully determine the best balance of classification confidence level and meaningful quality improvement. For example, a quality improvement program could use clinical performance as the initial evaluation framework (recognizing that higher confidence levels will result in a larger proportion of radiologist being classified in the “uncertain” zone), then further assess possible underperformers in an enriched environment with an artificially elevated event rate–in mammography, a higher proportion of cancers than expected in the clinical setting–for further evaluation and improvement monitoring (understanding the difficulties of emulating true performance accuracy in a test setting [36]).

We demonstrate that performance assessment errors are much more likely for CDR than for RR because of low incidence of breast cancer–between 2–10 cancers per thousand women [37]. While the challenge of demonstrating statistical differences due to low event rate in cancer screening has been recognized in the context of clinical trials [10], [11], [37] and practice-level performance accuracy assessment [6], [15], we extend this cautionary theme to physician-level performance measurement and also provide an intuitive graphical solution to avoid misclassification based on insufficient volume.

Our method exists in the context of a growing body of literature that catalogues the challenges of identifying physician outliers [38] and advances methods to address these challenges [6], [11], [39]. Some prior literature evaluates whether a metric is accurate in establishing physician performance relative to other physicians using a technique called “reliability” (i.e. primarily evaluating variation within a pool of providers) [39]. Rather, we have chosen to use a benchmark population (the BCSC) to develop an absolute performance requirement against which we judge performance and associated measurement confidence.

We recognize that our choice of benchmark threshold values (at the 10^th^ and 90^th^ percentiles of the BCSC reference distributions) and confidence level (95%) is somewhat ad-hoc. We do not contend that this choice is “correct,” just reasonable and useful for illustrative purposes. Our choices might optimally be more or less strict depending on the values, financial resources, and workforce considerations of the health system or population. Perhaps a screening program might rather use a 99% (wider) confidence interval for observed performance values thereby creating a stricter standard for classifying someone as adequate or inadequate. This would result in more physicians being in the uncertain zone, which would require more data or some other type of review to determine if performance is adequate. Using an 80% (narrower) confidence interval for observed performance values would more easily classify someone as adequate or inadequate, with fewer physicians in the uncertain zone. The exact values prescribed are not the point of our manuscript. Our methodology is intended to support any reference distribution, selected benchmark threshold (or consensus-developed performance range), and confidence interval considered appropriate for a given screening program [15]. While our ultimate goal in this manuscript is to provide a method and graphical presentation that is intuitive to individual physicians in the pursuit of fair and accurate performance assessment, further work on thresholds for particular settings will be important.

Our method focuses primarily on the impact of volume and incidence on whether a physician should be classified as having adequate or inadequate performance based on an observed performance value from a finite sample of patients. Differences in patient population and specifically disease incidence may influence performance measures [40]. We do not emphasize the possible influence of differences in patient population or practice characteristics for individual physicians here because this was beyond the scope of our goals. However, for this very reason, we are careful to demonstrate that the individual physicians in our analysis were practicing in an environment similar to the BCSC (Table 2), which sampled a large cohort of physicians in a range of practice settings with diverse patient populations.

Our results establish a general method for classifying physicians performing screening studies based on comparing observed performance values to benchmarks. Our method enables confident assertion of adequate or inadequate performance for some individuals and prompts further data collection for others. For our example, screening mammography, we find that one year of data is likely not enough to accurately assess individual physician performance, except for particularly high volume readers. These conclusions likely will apply to other screening programs; therefore, caution is warranted when assessing screening performance measures, particularly at the physician-level. As healthcare enters an era of “pay-for-performance,” and scrutiny of individual physician performance increases [41], development of analytic methods and evaluation programs that consider the statistical variation of observed performance values for screening will help avoid erroneously penalizing or rewarding physicians.

## Supporting Information

Figure S1 **CDR performance estimates create a sawtooth appearance for the benchmark threshold because cancers detected must reflect whole numbers.** The continuity correction becomes negligible for N>3000 screening mammograms. Curves derived using the Poisson distribution illustrating the effect of the continuity correction.
(EPS)

Figure S2 **RR performance estimates create a sawtooth appearance but it becomes smoother sooner due to higher event rate.** The continuity correction can be safely ignored even for low N, because the recall rate (RR) is higher than the cancer detection rate (CDR). Curves derived using the Poisson distribution illustrating the effect of the continuity correction.
(EPS)

Materials S1 **Online Data Supplement: Statistical methodology.**
(DOCX)
