# Supplementary material for: Addressing the Challenge of Assessing Physician-Level Screening Performance: Mammography as an Example
Source: PLoS One. 2014 Feb 21;9(2):e89418. doi: 10.1371/journal.pone.0089418 (PMC3931752; doi:10.1371/journal.pone.0089418)
Supplement: Materials S1 — Online Data Supplement: Statistical methodology. (DOCX) [file pone.0089418.s003.docx]

Online Data Supplement: *Statistical methodology*

The Wilson score confidence interval method with continuity correction is used to compute the confidence intervals for the binomial proportion of detected cancers and recalls. The continuity correction might be omitted when the sample size is very large. Other commonly used methods for calculating confidence intervals and a comparison of their performance can be found in Newcombe.[[1](#_ENREF_1)]

Let $\hat{p}$ be the estimate of the performance value. It is a ratio where the denominator is the volume (*n*). Let $p_{L}$ and $p_{U}$ be the lower and upper confidence limits for the performance value. The equations for calculating these confidence limits satisfy the equations:

$$\frac{\hat{p}-p_{L}-1/2n}{\sqrt{p_{L}(1-p_{L})/n}}= Z_{1-0.05/2}$$

$$\frac{p_{U}-\hat{p}-1/2n}{\sqrt{p_{U}(1-p_{U})/n}}= Z_{1-0.05/2}$$

where $Z_{1-0.05/2}$ = 1.96 is the 1 – 0.05/2 (0.975) percentile of a standard normal distribution. The relationship between the performance metric and volume when the lower bound of the confidence interval is equal to the benchmark threshold is obtained by replacing the lower confidence limit ($p_{L}$) in the first formula with the benchmark threshold value. For CDR we use 2.4 per 1000 and obtain

$$\frac{\hat{p}-0.0024-1/2n}{\sqrt{0.0024(1-0.0024)/n}}= 1.96$$

or,

$$\hat{p} = 0.0024+1/2n+1.96\sqrt{0.0024(1-0.0024)/n}$$

This equation allows us to calculate $\hat{p}$ for any given *n* such that the *n*, $\hat{p}$ pair will be on the upper curved line in (Figure 1a) that separates the *n*, $\hat{p}$ pairs that fall in the confidently adequate area from those in the uncertain area. To obtain the other boundary curve for CDR we substitute the benchmark threshold value for the upper confidence limit ($p_{U}$) in the second equation to obtain

$$\frac{0.0024-\hat{p}-1/2n}{\sqrt{0.0024(1-0.0024)/n}}= 1.96$$

or,

$$\hat{p} = 0.0024-1/2n-1.96\sqrt{0.0024(1-0.0024)/n}$$

This equation defines the lower, curved line (Figure 1a) that separates the *n*, $\hat{p}$ pairs that fall in the uncertain area from those in the confidently inadequate area. Equations for the curves in the RR figure (Figure 1b) are obtained in the same manner although the lines and the confidently adequate and confidenly inadequate areas are reversed .

Since both RR and especially CDR are small proportions, we obtained coverage probabilities to assess any possible discrepancy between the nominal confidence interval and the actual coverage probability. Coverage probabilities for the Wilson 95% confidence intervals at the benchmark threshold of CDR = 0.0024 ranged from 94.03 to 96.94% when sample size varied between 500 and 4000 in increments of 500. At RR = 0.168, Brown et al. list *n* = 24 as sufficient for the coverage probability to exceed 93% [[2](#_ENREF_2)]. Therefore, our methods are robust to both small estimated proportions and possibly small volumes, in the sense that the confidence intervals on which we rely have coverage probabilities close to their nominal 95% value.

There are a number of possible limitations of our approach. First, since cancers are so rare, the variability about an observed CDR might more plausibly be characterized with a Poisson confidence interval than with a binomial one. Figure S1 and S2 show the effect of using a Poisson confidence interval for both CDR and RR. Unfortunately, inverting a Poisson confidence interval (which is essentially what Wilson did with the standard asymptotic [Wald] confidence interval) to find a rate given the limits of confidence intervals is not easily tractable. Second, we did not consider the discreteness inherent in CDR and RR. The smooth curves depicted throughout our paper are idealized versions of the the sawtooth pattern evident in the Poisson approximations (Figure S1 and S2). Third, our use of the Wilson method is solely based on its ease of use, although to its credit, it is frequently recommended for its good coverage probability and small sample behavior. Fourth, since RR and CDR are positively correlated (RR = CDR + false positives/total screens), we might consider deriving joint confidence regions (JCR) instead of marginal (“one at a time”) confidence intervals. As the correlation between CDR and RR increases, it is likely that adequate performance based on marginal CDR and RR confidence intervals may turn out to be inadequate due to their being outside the 95% JCR. Fifth, we have assumed that the “case mix” of women screened is uniform and that the benchmark threshold is applicable to all physicians. Sixth, we have not considered any possible improvements introduced by using a sequential approach, in which CDR and RR are computed in interim fashion to provide timely feedback to practitioners. Seventh, we computed two-sided confidence intervals; it might be argued that one-sided confidence regions might be preferable. In practice, we do not think any of the above limitations is likely to impact our results appreciably. While we believe our methods may be improved, our choices simply provide a good first approximation and proof of concept.

**Supporting Information Legends:**

Curves derived using the Poisson distribution illustrating the effect of the continuity correction. (Figure S1) CDR performance estimates create a sawtooth appearance for the benchmark threshold because cancers detected must reflect whole numbers. The continuity correction becomes negligible for N > 3000 screening mammograms. (Figure S2) RR performance estimates create a sawtooth appearance but it becomes smoother sooner due to higher event rate. The continuity correction can be safely ignored even for low N, because the recall rate (RR) is higher than the cancer detection rate (CDR).

**References**

1. Newcombe RG (1998) Two-sided confidence intervals for the single proportion: comparison of seven methods. Statistics in Medicine 17: 857-872.

2. Brown LD, Cai TT, Das Gupta A (2002) Confidence Intervals for a Binomial Proportion and Asymptotic Expansions. Annals Stat 30: 60-201.
